# The Cut-Off Value for Classifying Active Italian Children Using the Corresponding National Version of the Physical Activity Questionnaire

**DOI:** 10.3390/sports10040061

**Published:** 2022-04-14

**Authors:** Corrado Lupo, Gennaro Boccia, Alexandru Nicolae Ungureanu, Anna Mulasso, Paolo De Pasquale, Annamaria Mancini, Pasqualina Buono, Alberto Rainoldi, Paolo Riccardo Brustio

**Affiliations:** 1 NeuroMuscularFunction, Research Group, School of Exercise & Sport Sciences, University of Turin, 10143 Turin, Italy; gennaro.boccia@unito.it (G.B.); alexandru.ungureanu@unito.it (A.N.U.); anna.mulasso@unito.it (A.M.); paolo.depasquale@unito.it (P.D.P.); alberto.rainoldi@unito.it (A.R.); paoloriccardo.brustio@univr.it (P.R.B.); 2 Department of Medical Science, University of Turin, 10143 Turin, Italy; 3 Department of Clinical and Biological Sciences, University of Turin, 10143 Turin, Italy; 4 Department of Movement Sciences and Wellness (DiSMEB), University Parthenope, 80133 Naples, Italy; annamaria.mancini@uniparthenope.it (A.M.); pasqualina.buono@uniparthenope.it (P.B.); 5 CEINGE-Biotecnologie Avanzate, 80145 Naples, Italy; 6 Department of Neuroscience, Biomedicine and Movement, University of Verona, 37131 Verona, Italy

**Keywords:** active children, non-active children, physical activity, PAQ-C, moderate-vigorous activity, ROC scales, sensitivity, accelerometer

## Abstract

The present study aimed to determine a cut-off value following the filling in of a questionnaire (PAQ-C-It) to identify active Italian children. One-hundred-twenty-nine primary school children (5 Piedmont schools; 47.3% female; mean age = 10 ± 1 years) wore an accelerometer (Actigraph wGT3X-BT) to objectively quantify individual moderate-to-vigorous physical activity during one week. Afterwards, the PAQ-C-It was filled in by participants. A ROC curve procedure was applied to obtain an active/non-active cut-off point. Spearman’s correlation coefficient was also applied to establish the relationship between the two parameters. According to the ROC analysis, the PAQ-C-It cut-off point value is identifiable at >2.75 to indicate active children (area under the curve = 0.62; standard error = 0.05; *p* = 0.025; coefficient intervals = 0.518–0.716; sensitivity = 0.592, specificity = 0.382), determining that 65 participants (55%) were non-active (mean PAQ-C-It value = 2.3 ± 0.4; active mean PAQ-C-It value = 3.3 ± 0.4). Spearman’s correlation coefficient results were significant but with a small effect size (rho = 0.214; *p* = 0.008). In conclusion, the present results suggest that the PAQ-C-It can be cautiously used as tool to practically classify active Italian children because of a non-solid relationship between respective accelerometer data and MVPA daily data.

## 1. Introduction

Physical activity (PA) is considered a key predictor of physical and mental health in children and adolescents [1,2]. On the contrary, physically inactive behavior is harmful for health, causing short- and long-term negative health consequences like overweight/obesity and non-communicable diseases [3,4,5]. For this reason, children and adolescents should perform at least an average of 60 min/day of moderate-to-vigorous physical activity (MVPA) including sports and leisure activities (i.e., mostly aerobic) across the week [6]. However, two-thirds of children and adolescents spend a large part of their waking time on sedentary activities like television-viewing and computer use [7,8], resulting in low MVPA, especially in countries such as Italy [9], despite school-based policy initiatives promote PA to enhance healthy and active lifestyle [10,11,12,13].

In order to monitor and assess the adherence to MVPA, as well as PA in general, different measurement methods, both subjective (e.g., diaries/logs, self-reported questionnaires or interviews) and objective (e.g., accelerometers, heart rate monitors and pedometers) are applied [14,15]. Among the objective monitoring of PA, wearable devices with accelerometer technology allows for the conversion of recorded accelerations to quantifiable counts [15], providing information about intensity, frequency and duration of activities [14]. Although their validity was reported during free-living activities in youth [16], some limitations still exist. In particular, they are better at detecting low-intensity activity (e.g., walking and jogging) while they underestimate non-ambulatory actions such as cycling, swimming, and weight-lifting [17]. Moreover, regression equations used to extract MVPA scores from raw acceleration data may include systematic errors by determining cut-off points for different intensity levels [18,19]. However, the triaxial accelerometer is considered an appropriate tool for estimating MVPA compared to other expensive or non-practical (e.g., doubly-labelled water, indirect calorimetry, heart rate monitor and direct observation) measures [20].

Among self-report methods, PA questionnaires are practical to administer, cost-effective and allow an insight into the PA of a large-scale population [21]. In particular, in the pediatric population, the Physical Activity Questionnaire for children (PAQ-C) is widely used in research and field settings (e.g., school context) to discern general levels of PA over the last seven days in children aged 8–14 [14]. It seeks to assess PA through ten items, nine of which are used to calculate the final score by averaging the one to five ordinal scale (i.e., where a higher score indicates higher levels of activity) of the single items [22]. However, it can present some limitations, especially when administered to children [20]. Specifically, difficulties with understanding questions, recalling activities, misinterpretation or social desirability are more problematic with children that with adults, and they may affect the final score [20,23]. On one hand, the PAQ-C demonstrated an acceptable-to-good internal consistency, test–retest reliability, and sensitivity in detecting gender differences [20,22,24]. Nevertheless, correlation of PAQ-C with objective measurements as accelerometer scores, particularly with MVPA, is still an open question [14]. Indeed, while some studies reported a moderate correlation [21,24,25,26,27,28], others showed a low or no correlation [22,29,30,31,32] between the PAQ-C and the accelerometer scores. In particular, according to previous studies [26,27], low PAQ-C specificity may originate because of children’s potential overestimations or underestimations of the items compared to the accelerometer measurement, making the questionnaire potentially age-dependent. For this reason, the PAQ-C could be better adaptable to adolescents than children [30]. Nevertheless, it was suggested that the PAQ-C may be valid for identifying children’s PA behavior rather than for absolute PA [14].

Furthermore, when validating concurrent validity, the new assessment should be compared to a well-established measure. Nonetheless, the calibration process of the accelerometers through the definition of count thresholds and equations to process raw information led to a high variability in comparing the results of different research studies [33,34]. Moreover, when considering concurrent validity for self-reported tools, such as PAQ-C, and objective tools, such as accelerometers, cut-offs for estimating MVPA should also be defined along with simple correlations. Both arbitrary (i.e., based on age-sex-specific PAQ-score quartiles, age-sex-specific median split of PAQ score, or assigning PAQ scores ≤2 to “low activity,” >2 and ≤3 to “moderate activity” and >3 to “high activity”) [35,36,37] and accelerometer-based [30,38] cut-offs were proposed to categorize children’s PAQ scores and their respective PA level. In particular, PAQ-C score cut-off points were identified between 2.7 and 2.9, although they varied with respect to gender [38], age [30,38] and output variables from the accelerometers [30]. Specifically, the PAQ-C was more likely to discriminate between “active” and “non-active” Spanish children than the “steps/min” and the “accelerometer counts” variables [30]. In terms of sensitivity and specificity, PAQ-C cut-offs were reported to have a low capacity to identify the “non-active” children, although they were able to reveal the true negatives [30].

However, cut-offs for MVPA should be defined in accordance with specific populations. In fact, PA in children varies in relation to several demographic, biological, environmental, social and psychological factors [39]. Therefore, the aim of this study was to define PAQ-C-It score cut-off values for identifying active Italian children according to objectively measured MVPA data.

## 2. Materials and Methods

### 2.1. Participants

An invitation to participate in the study was sent to all parents who had their children and adolescents in five primary schools in the neighborhoods of Turin (northwest Italy). Sessions to provide preliminary information regarding the aim of the study to the children’s teachers and parents/guardians were held in all recruited schools. All schools/classes recruited for the study were part of the same institutional and regional office and therefore were receiving a similar delivery of educational curricula [12]. Parents/guardians and teachers provided written informed consent for participation in the study, according to the ethical standards provided in the 1964 Declaration of Helsinki. Measurements were applied between 13 April and 10 June 2021. Before data collection, the institutional review board of the University of Turin approved this study (9 March 2020: Protocol #134691). One-hundred and forty-nine children gave their written informed consent after receiving detailed information about the aims and procedures of the study. From this sample, subjects with incomplete PA data (*n* = 23) were excluded. A final sample of 126 children (47.6% girls) participated in this study.

### 2.2. Procedures

#### 2.2.1. Anthropometric Parameters

Stature was measured using a portable stadiometer (Model 214; Seca, Hamburg, Germany) with an accuracy of 0.01 m. Body mass was measured using an electronic scale (Model 876; Seca, Hamburg, Germany) with an accuracy of 0.1 kg. Both stature and body mass were measured without shoes. Waist circumference was measured to the nearest 0.01 m midway between the lowest rib and the iliac crest with the participants in the standing position. The Body Mass Index (BMI) was calculated as body mass divided by height squared (kg·m^−^^2^).

#### 2.2.2. Triaxial Accelerometry

The Actigraph GT3X-BT accelerometer (Actigraph LLC, Pensacola, FL, USA) was used to assess PA objectively. The GT3X-BT is lightweight (27 g), compact (3.8 × 3.7 × 1.8 cm), and has a rechargeable lithium polymer battery. In this study, accelerometer data was sampled at 30 Hz and summarized over 10 s epochs. The GT3X-BT proved to be a reliable and valid tool for the assessment of different types of physical activities, despite the accelerometer being word on the hips [40]. Each participant wore the GT3X-BT on the non-dominant wrist, fastened with a wrist strap. Participants were asked to only remove the device when: sleeping, practicing water-based activities, and engaging in contact sports. Accelerometers were programmed to start recording at 2 p.m. of the same day on which they received the device (i.e., on Wednesdays) and to record activity for the following 6 days (i.e., on Tuesdays, at 8 a.m.). Verbal instruction and demonstration on how to wear the accelerometer were provided by researchers when children received the device [41]. A pre-initialized accelerometer was individually distributed to students at school, for one class a week. 

Actilife Software (version 6.13.4 Actigraph Corporation) was used to process the accelerometer data. Periods of ≥60 min of zero values, allowing for 2 min of non-zero interruptions, were defined as accelerometer “non-wear” time and were removed from the analyses. The first day of recording (i.e., from 2 p.m. to midnight) as well as the last one (i.e., the sixth; until 8 a.m.) were not included in the analysis. Only participants with ≥4 complete days, including one weekend day, were included [42]. A day was considered valid if it contained ≥10 h of wear time for weekdays and ≥8 h for weekend days considering different sleep patterns at weekends [43]. The sum of vector magnitudes (VM), defined as the combined measure of the three axes (i.e., $\sqrt{x^{2}+y^{2}+z^{2}}$) was used to compute PA level. According to a previous study [44], we considered the cut-points for non-active children in relation to the following threshold: VM < 305; for light-intensity PA (LPA), as VM 306–817; for moderate-intensity PA, as VM 818–1968; for vigorous-intensity PA, as VM > 1969; and for MVPA, as VM > 818 (all referred to 5 s epochs). Minutes of MVPA was considered for the analysis.

#### 2.2.3. Physical Activity Questionnaire for Older Children (PAQ-C-It)

Subjective PA was assessed using the PAQ-C-It [25] at the end of the triaxial accelerometry wearing period. This questionnaire consists of ten items (the tenth item is not considered for the final summary score), and collects information on participation in different types of activities and sports, effort during physical education classes, and activity during lunch, after school, evening, and at the weekend, during the past 7 days [14]. In particular, the first item is an activity checklist of common sports and games, whereas the next six ones assess PA in specific moments of the previous week: physical education lesson, recess, lunch time, right after school, in the evening and during the weekend. The eighth item asks which statement “describes you best for the last 7 days”, and the ninth item is a checklist for the frequency of PA over the previous week. The tenth item, out of the total score calculation, aims to ask children if they are sick or something precludes them from doing their regular PA. Each item is scored between 1 (low PA) and 5 (very high PA) and the average score denotes the final PAQ-C-It activity summary score; therefore, high scores indicate higher levels of PA. Although the Italian version of the PAQ-C-It differs from the original English version, especially for the activities reported in the first item, it reported acceptable to good internal consistency (Cronbach’s alpha values 0.70–0.83) [25]. However, for a better filling in of the questionnaire, an expert was constantly available to support children in understanding items. 

### 2.3. Statistical Analysis

Extreme outliers for outcome variables, defined as above or below 2 standard deviations from the mean, were identified and excluded from the analyses. Participants’ anthropometric characteristics and PAQ-C-It and MVPA were described in terms of means and standard deviations (SDs). Independent t-tests were conducted to determine whether there were statistically significant differences in anthropometric characteristics and outcome variables. The relationship between the PAQ-C-It and minutes of MVPA was investigated using Spearman’s rank correlation coefficients (rho). Correlations < 0.29 were considered weak, between 0.30 and 0.39 moderate, between 0.40 and 0.69, and those above 0.70 were considered “very strong” [45]. Finally, receiver operating characteristic (ROC) curves [45] were carried out to identify the optimal cut-off point for the PAQ-C-It and need to discriminate non-active from active children based on Actigraph data. In accordance with the WHO, we considered 60-min of MVPA per day as a valuable threshold to discriminate between active and non-active children [6]. We identified the cut-off points which equated to the coordinate yielding the greatest sum of specificity and sensitivity [46]. Accuracy of classification for each set of cut-points was evaluated by calculating weighted statistics, sensitivity, specificity, and area under the receiver operating characteristic (AUC) curve. An area of 1 represents perfect classification, whereas an area of 0.5 represents an absence of classification accuracy. ROC-AUC values of >0.90 are considered excellent, 0.80–0.89 good, 0.70–0.79 fair and <0.70 poor [47]. All analyses were performed using SPSS 27.0 (Chicago, IL, USA). The level of significance was set at *p* < 0.05.

## 3. Results

Approximately 15.4% of eligible students were included in the study. The characteristics of the entire sample and considering gender are described as means and standard deviations (SDs) in Table 1. 

**Figure 1:**
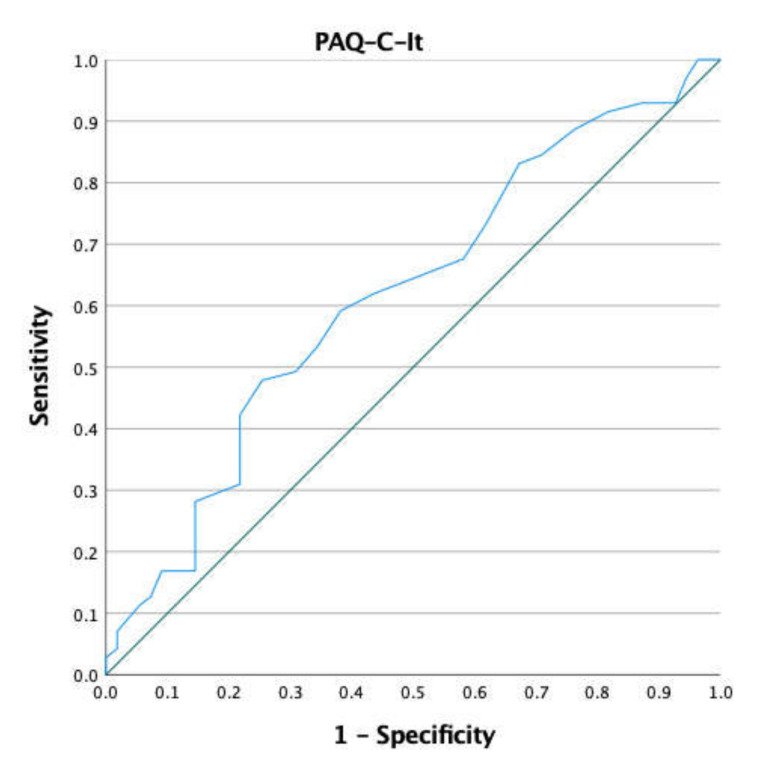
ROC curve of the PAQ-C-It and minutes of MVPA.

Briefly, no differences in terms of anthropometric characteristics were observed. Only for MVPA did females show a lower score than their gender counterparts (t = 2.34; *p* = 0.02; ES = 0.41; 95% CI = 0.06–0.77). A weak correlation (rho = 0.21; 95% CI = 0.03–0.37; *p* = 0.016) was found between the PAQ-C-It and minutes of MVPA. The plot of the ROC curve and the PAQ-C-It ROC curve coordinates (i.e., Sensibility, 1-Specificity and Youden Index) are reported in Figure 1 and Table 2, respectively. 

Considering 60 min of MVPA per day as an index of discrimination between active and non-active children, about 56% of the sample were considered active. Sensitivity and specificity were optimized at 2.75 points (sensitivity (59.2%; 95% CI: 47–71%) and 1-specificity (38.2%; 95% CI: 0.25–0.51)) with an associated AUC of 0.62 (95% CI: 0.52–0.72) and Youden index of 0.21. Based on the AUC, the classification accuracy was poor. The specificity value shows the low capacity of the PAQ-C-It to identify non-active children. For more details about statistical analysis, see Table 3.

## 4. Discussion

To distinguish between active and non-active individuals, PA cut-offs should be defined in accordance with several factors associated to the specific population [39]. Therefore, the present study aimed at defining PAQ-C-It score cut-off values using PA objectively and specifically measured on Italian children, in accordance with the international PA guidelines [6]. The main finding of this study was that PAQ-C-It could be utilized to identify active children (60 min of MVPA) using a cut-off point of 2.75, failing an accelerometer or other objective tool despite caution.

To our knowledge, this is the first study able to significantly define a PAQ-C-It cut-point value by accelerometry based on PA recommendations and with a device worn on the wrist. In fact, although our results reported a weak correlation between MVPA accelerometer and PAQ-C scores, this index could be considered useful for contributing to a better understanding of PAQ-C values. In addition, the ROC analyses reported a 2.75 PAQ-C-It cut-off score as a value to distinguish between Italian non-active and active children, which is the same threshold shown for co-aged Spanish children [30], in which children wore accelerometers on their hips. However, low specificity values have been observed in other studies [26,27], which may be due to a possible overestimation of the questionnaire measure compared to the accelerometer measurement or vice-versa. In the case of the present study, the results indicated that adolescents with lower levels of physical activity (<300 min/week) on the accelerometer were not classified in the same way by the questionnaire. Our results suggested that a cut-off of 2.75 points on the PAQ-C has a sensitivity of 59.2% in identifying true active children but a low capacity to find false active children (i.e., 38%). Thus, caution is necessary in any case when considering this cut-off. 

Although the present study, as well as the previous one with Spanish children of a similar age (1 year of difference), reported low sensibility and specificity values, and a poor AUC. The latter value for Italian children is significant and closer to the “fair” threshold. Nevertheless, these results should be considered with caution because the AUC should be >0.7 for an acceptable clinical. In fact, even though the PAQ-C-It is not associated with a clinical context, but with a public health one, it has anyway been associated with a poor validity (rho = 0.21, *p* < 0.05) for assessing MVPA in Italian children.

From a methodological point of view, we considered the cut-off points for non-active children according to the calibration procedure described in a previous study specifically focused on children of 8–12 years old who mounted the accelerometer on their non-dominant wrist [44]. Therefore, this characteristic should be considered in comparing results originating from different studies with adequate caution. This is the case in the already cited study on co-aged Spanish children [30], where MVPA data are associated with different calibration formulas and type of accelerometer wearing, despite the participants’ common age category. For this point, even though the hip has been the most common location, the wrist has been increasingly used during recent years in favor of better compliance in children [48], even considering that the latter modality is adequate in terms of validity, especially for high intensities of exercise [49]. However, the appropriate cut-off point depends on the aim of the identification. In fact, future studies exclusively recruiting active or non-active children could consider different cut-offs to benefit from a high specificity, thus guaranteeing a more solid identification of a specific children category. In addition, considering that it is not absolutely established that children performing structured physical activity (i.e., regular sport appointments) are also the most active ones, because of a sedentary lifestyle the rest of time, future studies could be performed for quantifying PA, taking count of the discrimination between children regularly performing sport activities or not. Finally, future studies could be also promoted for providing a different questionnaire where all items specifically refer to the daily PA practiced every day during the week and then summarize the score, preventing the recall of the duration and intensity of the events of the day, as well as for a replication on 12–14-year-old children of the same school to investigate eventual age-related effects.

The children who participated in the present study reported homogenous age, anthropometrics, and PAQ-C-It values between girls and boys, but a different mean MVPA level, where girls showed higher values. Besides a controversial trend with respect to previous studies [39,50], the combination of these results with PAQ-C-It values (for which boys reported a marginal higher level with respect to that of girls) leads to an overestimation of boys’, or an underestimation of girls’ individual PA evaluations, or even both trends. Therefore, a more homogenous sample in terms of MVPA values obtained by an accelerometer could contribute to a better solidity of the parameters associated with the validation of the first tool. In fact, regardless of the abovementioned methodological divergences, the Italian sample reported in the present study is characterized by a similar and general mean MVPA to the similar-aged Spanish children (Italian: 64.4 min/day; Spanish: 62.8 min/day) [30], but very different values between female children (Italian children: 68.8 min/day; Spanish children: 61.3 min/day). This highlights a potential limitation in providing a solid PAQ-C validation, even considering the controversial homogeneity of the PAQ-C-It scores between genders which is at a lower absolute level with respect to that provided for the Spanish children in any case (Italian: 2.81; Spanish: 3.09).

Although the PA of the Italian children recruited in the present study can be classified as “moderate activity” according to the questionnaire scores for both genders [35,36,37], the MVPA parameter generally reported values absolutely above the general average for both genders [9,14]. No speculation can be easily provided for clearly interpreting the gender trend that emerged in this study. Despite this, a general positive effect on PA can be associated with the socio-cultural environment of schools, because they are in countryside areas, where PA can be better promoted than in a big city [51].

## 5. Conclusions

The present study was able to demonstrate that the PAQ-C-It can be used only with caution as tool to practically classify active Italian children because of a non-solid relationship between respective accelerometer data and MVPA daily data. As a consequence, it could be partially considered only for those cases in which it is not possible to measure children’s PA by means of an accelerometer or other objective tools.

## Figures and Tables

**Table 1:** Characteristics of the participants (mean ± SD).

	All (*n* = 126)	Girls (*n* = 60)	Boys (*n* = 66)
Age (years)	9.9 (0.4)	9.9 (0.3)	9.9 (0.4)
Height (cm)	140.0 (6.5)	139.7 (5.8)	140.3 (7.0)
Weight (kg)	35.9 (7.7)	34.8 (7.6)	37.0 (7.6)
Waist circumference (cm)	63.5 (9.4)	60.3 (10.1)	66.2 (7.9)
BMI (kg·m^−2^)	18.2 (3.0)	17.7 (2.9)	18.7 (3.0)
PAQ-C-It score	2.81 (0.66)	2.77 (0.66)	2.83 (0.68)
MVPA (min/day)	64.4 (20.3)	68.8 (19.3) *	60.4 (20.6)

BMI: Body Mass Index; PAQ-C-It: Physical Activity Questionnaire for Children in Italy; MVPA: Moderate to Vigorous Physical Activity; *: significant (*p* < 0.05) differences between girls and boys.

**Table 2:** PAQ-C-It ROC Curve Coordinates (i.e., Sensibility, 1-Specificity and Youden Index).

Positive if Greater Than	Sensibility	1-Specificity	Youden Index
0.1	1	1	0
1.65	1	0.964	0.03
1.75	0.972	0.945	0.02
1.85	0.93	0.927	0.00
1.95	0.93	0.873	0.05
2.05	0.915	0.818	0.09
2.15	0.887	0.764	0.12
2.25	0.845	0.709	0.13
2.35	0.831	0.673	0.15
2.45	0.732	0.618	0.11
2.55	0.676	0.582	0.09
2.65	0.62	0.436	0.18
2.75	0.592	0.382	0.21
2.79	0.535	0.345	0.19
2.85	0.493	0.309	0.18
2.95	0.479	0.255	0.22
3.05	0.423	0.218	0.20
3.15	0.366	0.218	0.14
3.25	0.31	0.218	0.09
3.35	0.282	0.145	0.13
3.45	0.225	0.145	0.08
3.55	0.169	0.145	0.02
3.65	0.169	0.091	0.07
3.75	0.127	0.073	0.05
3.85	0.113	0.055	0.05
3.95	0.07	0.018	0.05
4.05	0.042	0.018	0.02
4.15	0.028	0	0.02
4.3	0.014	0	0.01
5.4	0	0	0

Note: Youden Index = sensitivity + specificity −1.

**Table 3:** ROC Curve Details Analyzing PAQ-C-It Score for WHO PA Recommendations.

	PAQ-C-It Score for MVPA (60-min)
AUC (95% CI)	0.62 (0.52 to 0.72)
Standard Error	0.05
*p*	0.025
Youden Index	0.21
Cut-off point	>2.75
Sensitivity (95% CI)	0.592 (0.47 to 0.71)
Specificity (95% CI)	0.382 (0.25 to 0.51)
Positive likelihood ratio (95% CI)	1.55 (1.05 to 2.28)
Negative likelihood ratio (95% CI)	0.66 (0.46 to 0.93)
Positive predictive values (95% CI)	66% (55% to 78%)
Negative predictive values (95% CI)	54% (41% to 66%)

AUC: Area under the curve.
